# Photoaging: UV radiation-induced inflammation and immunosuppression accelerate the aging process in the skin

**DOI:** 10.1007/s00011-022-01598-8

**Published:** 2022-06-24

**Authors:** Antero Salminen, Kai Kaarniranta, Anu Kauppinen

**Affiliations:** 1 Department of Neurology, Institute of Clinical Medicine, University of Eastern Finland, P.O. Box 1627, 70211 Kuopio, Finland; 2 Department of Ophthalmology, Institute of Clinical Medicine, University of Eastern Finland, P.O. Box 1627, 70211 Kuopio, Finland; 3 Department of Ophthalmology, Kuopio University Hospital, P.O. Box 100, 70029 Kuopio, Finland; 4 School of Pharmacy, Faculty of Health Sciences, University of Eastern Finland, P.O. Box 1627, 70211 Kuopio, Finland

**Keywords:** Aging, Anti-inflammatory, Carcinogenesis, Lifespan, UVA, UVB

## Abstract

**Background:**

Excessive exposure of the skin to UV radiation (UVR) triggers a remodeling of the immune system and leads to the photoaging state which is reminiscent of chronological aging. Over 30 years ago, it was observed that UVR induced an immunosuppressive state which inhibited skin contact hypersensitivity.

**Methods:**

Original and review articles encompassing inflammation and immunosuppression in the photoaging and chronological aging processes were examined from major databases including PubMed, Scopus, and Google Scholar.

**Results:**

Currently it is known that UVR treatment can trigger a cellular senescence and inflammatory state in the skin. Chronic low-grade inflammation stimulates a counteracting immunosuppression involving an expansion of immunosuppressive cells, e.g., regulatory *T* cells (Treg), myeloid-derived suppressor cells (MDSC), and regulatory dendritic cells (DCreg). This increased immunosuppressive activity not only suppresses the function of effector immune cells, a state called immunosenescence, but it also induces bystander degeneration of neighboring cells. Interestingly, the chronological aging process also involves an accumulation of pro-inflammatory senescent cells and signs of chronic low-grade inflammation, called inflammaging. There is also clear evidence that inflammaging is associated with an increase in anti-inflammatory and immunosuppressive activities which promote immunosenescence.

**Conclusion:**

It seems that photoaging and normal aging evoke similar processes driven by the remodeling of the immune system. However, it is likely that there are different molecular mechanisms inducing inflammation and immunosuppression in the accelerated photoaging and the chronological aging processes.

## Introduction

Repeated excessive exposures to UV radiation (UVR) induce alterations in the skin which have many similarities with those observed during chronological aging. This accelerated aging process has been called photoaging [1–3]. UVR, especially UVB, can damage DNA and protein structures in the cells of the skin, particularly in the epidermis. The inhibition of contact hypersensitivity by UVR exposure was a seminal observation indicating that UVR stimulates immunosuppression in the skin [4]. Subsequently, it was revealed that the UVR-induced stress triggers a local inflammatory state in the skin [5, 6]. Currently it is known that the UVR-induced inflammation in the skin stimulates a counteracting immunosuppression involving the expansion of immunosuppressive cells, especially regulatory *T* cells (Treg) [7–9]. The activation of immunosuppressive cells, such as Tregs, myeloid-derived suppressor cells (MDSC), and regulatory dendritic cells (DCreg), are able to expand the suppressive state occurring in the skin to the systemic immunosuppression in the immune system. Interestingly, chronological aging involves similar immune phenomena as photoaging, such as a chronic low-grade inflammation and a counteracting immunosenescence induced by immunosuppressive cells [10–12]. It seems that it is a remodeling of the immune system that is driving both the chronological aging and photoaging processes although the fundamental molecular mechanisms stimulating inflammation are most probably different. We will examine in detail the well-known processes inducing the activation of immunosuppressive state in photoaging to compare to an increase in immunosuppressive activity occurring in the normal aging process.

## Chronological aging of the skin

There are several extensive review articles which have elucidated the hallmarks of the normal, chronological aging process of the skin [13–15]. Clear histological changes involve a thinning of the skin which is caused by the atrophy of epidermal cell layers and the reduction of fibroblasts and the components of extracellular matrix (ECM) in the dermal layers. For instance, the amount of collagen decreases and it becomes fragmented and coarsely deposited. This is attributable to an increased secretion of matrix-degrading metalloproteinases by fibroblasts with aging [16]. In addition, there is an age-related decrease in elastic fibers as well as changes in the levels of glycosaminoglycans and proteoglycans in the dermis [17]. Elastin fibers become increasingly degraded with aging and the release of bioactive elastin-derived peptides is associated with many pathological conditions of skin [18]. The alterations in the amount of ECM components result in many clinical features encountered in the aged skin, such as wrinkles and loss of skin elasticity. The formation of wrinkles is associated with the age-dependent changes in the subcutaneous white adipose tissue (sWAT), under the dermal layers [19]. Zhang et al. [20] demonstrated that an age-related increase in TGF-β signaling enhanced the differentiation of mouse adipogenic dermal fibroblast (dFB) to pro-fibrotic cells, thus reducing dermal fat as well as decreasing the antimicrobial immunity of the skin. An inhibition of TGF-β receptor (TGFBR) signaling was claimed to enhance the adipogenic potential of dFBs and increase the resistance to skin infections. There are studies indicating that this dermal trans-differentiation process is driven by the age-related inflammation in the sWAT [19]. An increase in anti-inflammatory TGF-β signaling might be associated with the accumulation of immunosuppressive cells with aging within the dermis [21, 22].

An accumulation of senescent cells within tissues is a common hallmark of the aging process [23]. Different cell types in the skin, such as keratinocytes, fibroblasts, melanocytes, and stromal stem cells, can express many of the markers of cellular senescence, e.g., p16INK4a and SA-β-galactosidase [22, 24–26]. Interestingly, senescent cells display a pro-inflammatory phenotype since they secrete diverse cytokines, chemokines, and matrix metalloproteinases (MMP). This state has been termed the senescence-associated secretory phenotype (SASP) [27]. A number of disturbances occurring in the skin with aging can trigger a senescent state, e.g., DNA damage and telomere shortening, oxidative stress, endoplasmic reticulum stress, and mitochondrial and energy metabolic deficiencies [28]. There is convincing evidence that pro-inflammatory senescent cells enhance chronic low-grade inflammatory phenotype with aging in many tissues. This state has been called inflammaging [10]. Ruhland et al. [22] demonstrated that senescent stromal cells in mouse skin clearly increased the number of senescent cells within skin stroma. They also revealed that senescent cells promoted the development of local inflammatory microenvironment in mouse skin. Several studies have reported that the aging process is connected with a low-grade inflammaging in the skin of both mice and humans [22, 29]. Aging of the skin also involves a decrease in the numbers of stem cells and Langerhans cells which are the antigen-presenting cells of the skin. Moreover, the pro-inflammatory changes in tissue-resident macrophages enhance inflammaging in the skin [29]. Interestingly, Ruhland et al. [22] revealed that a senescence-induced inflammation was associated with an increase in the numbers of immunosuppressive Tregs and MDSCs in mouse and human skin. Several other studies have also demonstrated that Tregs accumulate within the skin during the aging process [21, 30]. It seems that inflammatory changes stimulate a counteracting immunosuppression intended to protect skin from excessive inflammatory injuries. Immunosuppressive cells secrete anti-inflammatory cytokines, such IL-10 and TGF-β, which not only are able to decrease the dermal adipose tissue [20] but they can also promote cellular senescence and disturb the structures of ECM (see below).

## Photoaging: an accelerated aging process of the skin

Repeated excessive exposures to sunlight or the light of UV lamps evoke alterations in the skin which are reminiscent but not identical to those observed during the normal aging process. Several review articles have compared the pathophysiological properties of chronological aging and photoaging [3, 31] “Conclusions”. Solar radiation spectrum (from 290 to 1 mm) contains ultraviolet radiation, both the UVB (280–320 nm) and the UVA (320–400 nm), visible light (400–700 nm), and infrared radiation (over 700 nm). UVB accounts for 5% and UVA for 95% of the terrestrial UV radiation of sunlight. The penetration of radiation in the skin is dependent on its wavelength, i.e., UVB radiation penetrates only into epidermis, UVA radiation into the dermis, while visible and infrared radiation can reach the subcutaneous adipose tissue [32]. UVR damages the skin, whereas visible and infrared radiations can induce both beneficial and deleterious responses in the skin [33, 34]. UVR targets molecular structures, called chromophores within the skin, eliciting significant stress responses and activating the immune system. UVB exposure can induce ferroptosis in the keratinocytes of human skin and thus trigger their death [35]. Increased wrinkles and spider veins, epidermal atrophy, increased melanogenesis and irregular pigmentation, and even sunburns are common clinical phenotypes of photoaging [2]. Major alterations appear within the dermis although there is a marked decline in the amount of subcutaneous fat [36], probably caused by the TGF-β-induced fibrotic transformation of adipocytes (see above).

The wavelength and dose of the radiation, e.g., sunlight, have an important role in shaping the UVR-induced responses. It is known that both UVB and UVA cause damages in the skin, e.g., its DNA as well as the ECM, and subsequently induce immunosuppression [37, 38]. The peak wavelength for the local immunosuppression by UVB exposure is at 300 nm in human skin, whereas it is at 370 nm for UVA radiation [39, 40]. Given that sunlight contains a much greater amount of UVA than UVB radiation, the relative solar immunosuppressive response is threefold higher than that of UVB [40]. Poon et al. [41] demonstrated that there was a significant interaction between the UVB and UVA radiation of sunlight in the generation of immunosuppression in human skin, i.e., UVB induced an earlier intensive response, whereas UVA affected more slowly. It seems that UVB and UVA radiations evoke similar mechanisms to induce inflammation and subsequently immunosuppression in the skin. For instance, UVA irradiation is also able to damage DNA and trigger oxidative stress in the skin [42, 43]. However, there are reports indicating that UVA radiation can induce a photoimmune protection against the UVB-induced immunosuppression [44, 45]. Reeve and Tyrrell [44] demonstrated that this adaptive response was dependent on the increase in the expression of heme oxygenase (HO) evoked by UVA radiation. It was claimed that the UVA-induced induction of HO prevented the UVB-induced immunosuppression by inhibiting oxidative stress [46].

Nuclear and mitochondrial DNA, are important targets of UVR, both UVA and UVB radiation [47–49]. UVR is able to damage DNA either directly by forming pyrimidine dimers or indirectly by stimulating oxidative stress which oxidizes guanine bases generating 8-oxo-7,8-dihydroguanine (8-oxoG). The 8-oxoG modification is a hallmark of aging tissues and age-related diseases [50, 51]. Interestingly, DNA damage is a potent inducer of inflammatory responses via different pathways [52–54]. Given that inflammation can also trigger DNA damage, it seems that UVR exposure can create a vicious cycle [55] which is a possible source of the photoaging process in the skin. Yamada et al. [56] demonstrated that there was a significant decline in the ability to remove the UVB-induced pyrimidine dimers from the epidermis in the skin of elderly people, possibly enhancing the accumulation of DNA damages. Given that DNA damages are a major enhancer of cellular senescence [57], the elimination of 8-oxoG prevented the rapid onset of cellular senescence in human skin fibroblasts [58]. An increased accumulation of senescent cells is associated with the photoaging process [59]. Moreover, it is known that senescent cells can contribute to the senescence of neighboring cells through the bystander effect involving ROS compounds, cytokines, and MMPs [60, 61]. Widel et al. [62] demonstrated that both UVA and UVB radiation-induced apoptosis and cellular senescence as a bystander effect in human dermal fibroblasts through the secretion of ROS compounds and pro-inflammatory cytokines. It seems that bystander effects have a significant role in the expansion of photoaging in the skin.

UVR also stimulates the degradation of dermal ECM components, e.g., collagen, elastin, and glycoproteins [63]. UVR induces collagen fragmentation and aggregation, i.e., similar changes as encountered in the intrinsic aging process but much faster than in normal aging [63, 64]. UVR exposure significantly increased the expression and secretion of matrix metalloproteinases which induced the degradation of ECM proteins [65]. In contrast, an increased expression of elastin and its splice variant clearly augmented the dermal accumulation of elastin and its fragments in the photoaged skin, a process called solar elastosis [66, 67]. Pathological alterations in the components of ECM have been reported to promote tissue fibrosis and cellular senescence as well as triggering inflammatory reactions [68, 69]. Some elastin and collagen fragments can act as matrikines and stimulate inflammatory responses [70]. In summary, UVR directly and indirectly disturbs the skin’s homeostasis, inducing cellular stresses, such as oxidative, endoplasmic reticulum, and mitochondrial stresses [47, 71, 72]. There is convincing evidence that UVR exposure aggravates the mechanisms capable of enhancing chronological aging in the skin. Given that changes in the immune system have a key role in the chronological aging process, we will next focus on the immune mechanisms which are driving the photoaging process in the skin.

## Immune mechanisms driving photoaging process in the skin

### UVR stimulates inflammation

**Fig. 1:**
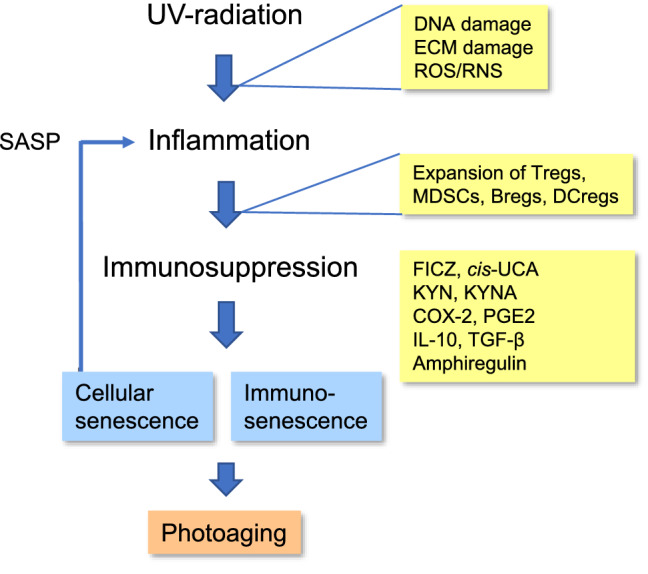
The pathogenesis of UVR-induced photoaging in the skin. UVR exposure induces damages in DNA and ECM of the skin. UVR also enhances the generation of ROS/RNS compounds and thus it promotes oxidative stress. UVR-induced alterations elicit inflammatory state in the skin. Subsequently, inflammation stimulates the expansion of immunosuppressive cells in the skin, thus counteracting the inflammatory state. The expansion of Tregs, MDSCs, Bregs, and DCregs enhances immunosuppressive activity in the skin. There are several mechanisms which can evoke the immunosuppressive state in the skin: (i) UVR stimulates the generation of FICZ and cis-UCA compounds, (ii) inflammation stimulates the synthesis of KYN and KYNA, (iii) inflammation activates COX-2 and increases the generation of PGE2, (iv) immunosuppressive cells secrete anti-inflammatory cytokines, such as IL-10 and TGF-β, and (v) immune cells secrete amphiregulin which exerts a suppressive activity on Tregs. The activation of UVR-inflammation-immunosuppression pathway promotes the senescence of immune and non-immune cells in the skin. Senescent cells express a pro-inflammatory secretory phenotype (SASP) which is driving the pathological alterations evident in the skin. Chronic inflammation and the counteracting immunosuppression cause degenerative alterations in the skin inducing the photoaging state. *Breg* regulatory *B* cell, *COX-2* cyclo-oxygenase-2, *DCreg* regulatory dendritic cell, *ECM* extracellular matrix, *FICZ* 6-formylindolo[3,2-b]carbazole, *IL-10* interleukin-10, *KYN* kynurenine, *KYNA* kynurenic acid, *MDSC* myeloid-derived suppressor cell, *PGE2* prostaglandin E2, *SASP* senescence-associated secretory phenotype, *TGF-β* transforming growth factor-β, *Treg*, regulatory *T* cell, *UCA* urocanic acid, *UVR* ultraviolet radiation

A chronic low-grade inflammation is associated with the photoaging process in the skin [5, 6, 73]. Moreover, the long-term presence of inflammatory microenvironment enhances the risk for carcinogenesis and metastasis. As discussed earlier, the UVR-induced DNA damage and alterations in ECM disturb homeostasis and trigger cellular stresses which activate inflammatory responses in the skin (Fig. 1). The NF-κB and p38MAPK pathways are the major inducers of inflammatory responses, both in non-immune cells and immune cells of the skin. There is convincing evidence that UVB radiation also evokes inflammation by activating inflammasomes [74–76]. Hasegawa et al. [75] demonstrated that DNA damage in human keratinocytes activated NLRP3 inflammasomes promoting the secretion not only IL-1β but also other inflammatory factors, e.g., IL-1α, IL-6, and TNF-α. Interestingly, the UVR-induced stress in human skin triggers the generation of senescent cells which display the pro-inflammatory SASP state [59, 77]. Premature senescence can appear in keratinocytes, fibroblasts, melanocytes, and subcutaneous preadipocytes [78]. The senescence of cells arrests their proliferation and stimulates the expression of a large number of pro- and anti-inflammatory mediators [27, 79]. Interleukins, chemokines (e.g., CCL2, CCL3, CXCL1, CXCL8), and colony-stimulating factors (GM-CSF) are major secreted pro-inflammatory factors detected in many experimental models. Kondo [80] reviewed the results from early biochemical studies on skin photoaging indicating that photoaging stimulated the expression of many interleukins and chemokines in immune cells, e.g., monocytes, macrophages, and natural killer (NK) cells, as well as in several non-immune cells. Subsequently, genome-wide transcriptional profiling studies examining the UVB-induced changes in human skin have revealed a strong increase in the expression levels of many cytokines and especially of several chemokines (CCL3, CXCL1, CXCL3, and CXCL5) [81]. In addition, the robust increase in the expression of COX-2 indicates that prostanoids have a role in the photoaging process [81, 82]. It seems that the UVR-induced damages stimulate skin defense by activating the cytokine-induced transcriptional responses and the chemokine-controlled recruitment of immune cells into affected skin.

Three different pathological phases are evident after skin has been exposed to UVR [83, 84]. The early vasodilatory phase involves an increased blood flow, erythema, dermal edema, mast cell degranulation, and pain sensitivity. In the next inflammatory phase, skin becomes infiltrated by neutrophils, monocytes, and *T* cells. This phase also involves an increased expression and secretion of pro-inflammatory cytokines and other inflammatory mediators. The third phase, called the regressive or resolution phase, contains many counteracting responses to the acute inflammation. The resolution phase comprises many anti-inflammatory events, such as the recruitment and expansion of immunosuppressive cells in the affected skin, as well as the secretion of anti-inflammatory cytokines, such as IL-4, IL-10, TGF-β. There are studies indicating that the resolution of acute inflammation triggers a prolonged immunosuppressive post-resolution phase coexisting with the repair processes in the inflamed tissue [85, 86]. Interestingly, UVR treatment induces a clear increase in the expression and secretion of IL-10 and TGF-β cytokines in human keratinocytes [87–89] as well as there appears an infiltration of IL-10-positive neutrophils and macrophages into human skin [90, 91]. Debacq-Chainiaux et al. [89] reported that a repeated exposure to UVB evoked a TGF-β-driven premature senescence in human dermal fibroblasts. Currently, it is known that UVR induces a profound remodeling of the immune system in the skin and prolonged inflammatory and immunosuppressive states might induce a photoaging of the skin.

### UVR-induced immunosuppression

The skin is not only a physical barrier between the environment and the internal organs but it is also an immune organ which contains different immune-competent cells, e.g., Langerhans cells, macrophages, mast cells, dendritic cells, dendritic epidermal *T* cells, Treg cells, and in addition, keratinocytes and melanocytes also possess their own distinctive immune properties [92, 93]. Recent studies have revealed that the subsets of resident and migrant Treg cells have a significant role in the maintenance of skin homeostasis, such as in the resolution of skin inflammation, wound healing, and immune tolerance to commensal microbes [94–96]. The resident Treg cells of human skin are a dynamic and heterogeneous population of the FoxP3-containing *T* cells. Sanchez Rodriguez et al. [97] reported that about 20% of CD4 *T* cells were the FoxP3-positive Treg cells in adult human skin. The skin-resident Tregs displayed antigen-specific properties and they became strongly expanded under inflammatory conditions, e.g., in psoriasis [97]. Ikebuchi et al. [98] revealed the high functional diversity of Tregs in mouse inflamed skin containing two major populations, i.e., the resident Treg cells and the bidirectional migratory population including Tregs migrating from the skin to lymph nodes and back to the skin. Tomura et al. [99] have also described a bidirectional trafficking of Tregs between the skin and the draining lymph nodes. Interestingly, the resident Tregs extensively expressed CD25 and CD39 proteins, common markers of immunosuppressive cells [100, 101]. There is convincing evidence that the numbers of Tregs increase in mouse and human skin during the aging process [21, 22, 30]. Tregs are the major immunosuppressive cells which suppress the functions of innate and adaptive immune cells. For instance, they inhibit inflammatory responses but they can also provide a means for cancer cells to evade immune surveillance [102–104]. There are also observations indicating that a decline in cutaneous delayed hypersensitivity with aging can be attributed to an accumulation of immunosuppressive Tregs [21, 30].

Over 30 years ago, it was observed that UVR exposure was able to induce immunosuppression and promote skin carcinogenesis [4, 105]. These studies provided the ground for photoimmunology. The contact hypersensitivity model was exploited in mice to reveal the local and systemic immunosuppression triggered by repeated exposures to UVR [106, 107]. Noonan et al. [106] demonstrated that the immune suppression was attributed to defects in antigen presentation in mice. Interestingly, it was also revealed that UVR treatment induced a systemic immunosuppression, i.e., the sensitivity of the skin to insults was reduced at locations far from the irradiation site [107]. Moreover, Harriott-Smith and Halliday [108] demonstrated that the passive transfer of serum from the UV-irradiated mice into non-treated mice suppressed the contact hypersensitivity in their skin. Many subsequent studies have revealed that UVR exposure of the skin induced both local and systemic activation of the immunosuppressive network [4, 7–9]. In particular, UVR treatments robustly increased the expansion and activity of Treg cells in the affected skin (Fig. 1). There are several studies indicating that it is the skin-specific DCs that affect the properties of Tregs in the UV-treated skin [109, 110]. Schwarz et al. [109] reported that after UVR treatment Langerhans cells (LC) emigrated into regional lymph nodes where they presented the antigen to Tregs and programmed their homing into the affected skin. Once they reached the skin, the Tregs secreted IL-10 and induced a general immunosuppression [4]. Recently, Yamazaki et al. [110] demonstrated that UVB exposure activated the dermal Langerin (−) DCs to increase their expression of CD86 protein, a co-stimulatory ligand for the suppressive activity of Tregs. The activation of Langerin (−) DCs augmented the expansion and immunosuppressive activity of FoxP3 Tregs in the UVB-exposed skin. Moreover, it is known that UVR treatment stimulated the recruitment of immunosuppressive MDSCs into the UV-exposed sites in mouse skin [111, 112]. UVR exposure also increased the occurrence of immunosuppressive DCregs, regulatory *B* cells (Breg), and natural killer *T* (NKT) cells in the affected skin [113–115]. Given that this immunosuppressive network co-activates the suppressive functions of its members, the immunosuppressive phenotypes of DCreg, Breg, and NKT cells might be induced by the secretion of IL-10 and TGF-β from Tregs and MDSCs.

The UVR-induced immunosuppression reduces the functional capacity of both the local and systemic immune system [1, 116, 117]. Concurrently with the increased differentiation of Tregs after UVR exposure, there was a decrease in the proliferation of mouse effector CD4 and CD8 *T* cells both in the skin and the skin-draining lymph nodes [118]. Li-Weber et al. [119] demonstrated that the UVR exposure of human *T* cells inhibited their activation by blocking the TCR-mediated stimulation of ERK and NF-κB signaling. UVR treatment also inhibited the production of cytokines induced by TCR stimulation. It is known that UVR-induced immunosuppression significantly reduced the antigen-presenting capacity of human Langerhans cells and dermal DCs [116, 120, 121]. There is also a close crosstalk between DCs and Tregs. For instance, UVB exposure enhanced the maturation of dermal DCs which subsequently evoked the expansion of Tregs in mouse skin [110]. On the other hand, UVR-induced Tregs switched mouse DCs from displaying a stimulatory phenotype to becoming immunosuppressive DCregs [122]. Moreover, Neill et al. [123] demonstrated that UVB treatment suppressed the cytotoxic activity of natural killer (NK) cells but not that of cytotoxic CD8 T cells. It seems that UVR exposure might impair the immune surveillance of viruses and cancer cells and thus enhance carcinogenesis. It is known that UVR exposure evoked the infiltration of monocytes from the blood into the inflamed dermis and subsequently monocytes differentiated to macrophages [1]. Later in the course of inflammation, macrophages adopted the properties of M2 anti-inflammatory macrophages. These studies indicate that the functional properties of the UVR-remodeled immune cells are reminiscent of those encountered in chronic inflammation-induced immunosenescence which has been associated with the aging process and other long-lasting inflammatory conditions [124].

Currently the molecular mechanisms and signaling pathways which stimulate the UVR-induced immunosuppression still need to be clarified. It is known that chronic inflammation is a potent inducer of immunosuppression but UVR exposure generates immunosuppression much faster than common inflammatory states although inflammation seems to have a crucial role in the photoaging process. Interestingly, UVB radiation directly targets the amino acid L-tryptophan in cytoplasm processing it to 6-formylindolo[3,2-b]carbazole (FICZ) [125, 126]. FICZ is an activating ligand of aryl hydrocarbon receptor (AhR) which regulates many immunosuppressive activities, e.g., FICZ exposure stimulated the expression of COX-2 in human keratinocytes [125]. The COX-2/PGE2 signaling evoked the activation of PGE2 receptor 4 (EP4) which induced the generation of UVR-mediated Tregs and consequently promoted a systemic immunosuppression in mice [127]. UVB exposure also stimulated the kynurenine (KYN) pathway in human fibroblasts and keratinocytes [128]. It is known that KYN and kynurenic acids (KYNA) are inducers of AhR signaling and subsequently they can promote Treg generation [129, 130]. Navid et al. [131] reported that UVR exposure induced the generation of Tregs in an AhR-dependent manner in mouse skin. Moreover, Bruhs et al. [132] demonstrated that the UVR-induced activation of the AhR factor switched antigen-presenting DCs from a stimulatory state into the regulatory (DCreg) phenotype which consequently promoted the differentiation of Tregs. There is substantial evidence that a chronic activation of AhR signaling in the skin promotes both premature aging and carcinogenesis by affecting energy metabolism, ECM structures, DNA repair, and apoptosis [133].

The UVR-induced immunosuppression has been under intensive research for years since UVR is an important inducer of both skin cancers and photoaging. In the epidermis, urocanic acid (UCA) effectively absorbs UV irradiation triggering the isomerization of cutaneous *trans*-UCA to *cis*-UCA which promotes the development of immunosuppression [134, 135]. Walterscheid et al. [136] reported that *cis*-UCA treatment reduced the UVR-induced delayed-type of hypersensitivity in mice by binding to one subtype of serotonin receptors, i.e., 5-hydroxytryptamine 2A (5-HT2A) receptors. Recently, Korhonen et al. [76] demonstrated that *cis*-UCA exposure prevented the UVB-induced inflammasome activation in human corneal epithelial cells (HCE-2). *cis*-UCA treatment inhibited the secretion of IL-1β, IL-6, and IL-8 from HCE-2 cells. This indicates that *cis*-UCA exerts an anti-inflammatory activity which might inhibit the inflammation-induced immunosuppression, e.g., through KYN-AhR signaling. There is clear evidence that UVB treatment was able to elevate the expression and secretion of the anti-inflammatory cytokine, IL-10, in human keratinocytes [87, 137]. Moreover, the UVB-induced Tregs robustly secreted IL-10, thus enhancing the bystander suppression of effector immune cells [9]. UVB treatment also increased the expression and secretion of TGF-β in human keratinocytes [88]. It is known that TGF-β can stimulate the expression of AhR which consequently increases the generation of Tregs [130]. Wang and Kochevar [138] demonstrated that UVB exposure increased the expression and activity of TGF-β in human keratinocytes in a ROS-dependent manner. Interestingly, they reported that UVB treatment induced the generation of ROS compounds by activating the EGF receptor (EGFR). Meulenbroeks et al. [139] reported that amphiregulin (AREG), an EGF-like growth factor, plays a crucial role in the UVB-induced, Treg-mediated immunosuppression in mouse skin. They revealed that the immunosuppression was dependent on the secretion of AREG from basophils in the UVB-treated skin. Wang et al. [140] demonstrated that AREG conferred suppressive functions on Tregs via EGFR/GSK-3β/FoxP3 signaling. The UVR-induced expansion of Tregs and the occurrence of immunosuppression prevent excessive inflammatory responses but if there is a chronic increase in immunosuppressive activity, this induces immunosenescence and thus disturbs homeostasis within the skin promoting the photoaging process.

### Chronic immunosuppression promotes degeneration of the skin

Immunosuppressive cells, such as Tregs, MDSCs, and DCregs, possess an armament of mechanisms which they exploit to suppress excessive inflammatory responses including (i) the secretion of anti-inflammatory cytokines, such as IL-10 and TGF-β, (ii) the release of ROS compounds, reactive nitrogen species (RNS), and PGE2, and (iii) the expression of amino acid catabolizing enzymes, e.g., indoleamine 2,3-dioxidase 1 (IDO1) and arginase 1 (ARG1) [141–143]. Nonetheless, there is convincing evidence that in persistent inflammatory states, these immunosuppressive tools exert harmful effects on both immune and non-immune cells in inflamed tissues. For instance, the immunosuppression induced by the excessive activation of Tregs in inflamed skin impairs the functions of several effector immune cells (see above). Similar defects in the functional properties of immune cells have been observed with aging and in many chronic inflammatory conditions [124]. This state has been called immunosenescence. Moreover, it is known that the activation of immunosuppressive cells increases cellular senescence of non-immune cells in host tissues, e.g., in the skin [22]. For instance, TGF-β treatments were capable of triggering cellular senescence in different cell types [144]. The activation of human Tregs disturbed the immunosurveillance and cytotoxic properties of NK and CD8 *T* cells [145, 146]. UVR exposure also suppressed the activities of NK cells in the skin (see above). The deficiency of the immune surveillance and cytotoxic capacity of NK and CD8 *T* cells increased the accumulation of senescent cells into affected tissues [147–149]. Subsequently, the SASP phenotype of senescent cells, i.e., the increased secretion of inflammatory mediators, enhanced the inflammatory state and the maintenance of an immunosuppressive condition in the skin (Fig. 1). It is known that this feed-forward regulation promotes the aging process and age-related diseases [149]. Recently, Fitsiou et al. [59] have reviewed an important role of the UVR-induced cellular senescence and the SASP phenotype in the generation of photoaging in the skin. The accumulation of senescent immune and non-immune cells within tissues is a hallmark of both the normal aging process and the accelerated photoaging.

Chronic inflammation and counteracting immunosuppression impair tissue homeostasis not only by increasing the accumulation of senescent immune and non-immune cells but also by inducing extensive bystander degeneration in inflamed tissues. We have recently reviewed the common degenerative changes induced by anti-inflammatory cytokines, ROS/RNS, and enhanced catabolism of L-arginine (L-Arg) and L-tryptophan (L-Trp) via the activation of ARG1 and IDO1 [143]. Briefly, an increased exposure to TGF-β, as induced by UVR, inhibited cell proliferation [150], enhanced tissue fibrosis [151], stimulated the expression and secretion of ECM remodeling enzymes, such as matrix metalloproteinases and collagenases [152], and even promoted the inflammation-associated myelopoiesis in the bone marrow [153]. Moreover, TGF-β signaling was able to remodel the chromatin landscape and thus enhance cellular senescence and the cardiac aging process [154]. Ke and Wang [155] have discussed some crucial effects of TGF-β signaling in photoaging, e.g., the inhibition of keratinocyte proliferation and the degradation of collagen and elastin fibers in photoaging. Accordingly, although IL-10 cytokine has an important role in IL-10-mediated anti-inflammatory responses, e.g., it can limit contact hypersensitivity in the skin [156], it is a pleiotropic factor which can elicit harmful effects in a context-dependent manner. For instance, IL-10 exposure inhibited the antigen presentation by antigen-presenting cells (APC) [157], thus inducing detrimental effects, e.g., in infections. Moreover, IL-10 cytokine inhibits autophagy in many cells [158], thus disturbing cellular proteostasis in inflamed tissues. The secretion of ROS/RNS is one of the suppressive mechanisms possessed by immunosuppressive cells, e.g., MDSCs. For instance, the secretion of ROS by MDSCs inhibited the TCR-mediated *T* cell activation [159]. ROS compounds also activated latent TGF-β cytokines in inflamed tissues and thus they not only augment anti-inflammatory potency [160] but they can also promote many of the pathological responses provoked by TGF-β. There is good evidence that oxidative stress has a significant role in the aging process [161], especially in the photoaging phenomenon [162].

Immunosuppressive cells exploit the catabolism of certain amino acids, such as L-Arg and L-Trp, to promote immunosuppression in inflamed tissues. Many immune effectors are auxotrophic for these amino acids and thus their depletion inhibits protein synthesis and subsequently suppresses cellular proliferation. Immunosuppressive cells are enriched with ARG1 and IDO1 enzymes which not only deplete L-Arg and L-Trp, respectively, but also generates immunoregulatory metabolites, i.e., ARG1 produces nitric oxide (NO) and polyamines, whereas IDO1 stimulates the KYN pathway [163, 164]. NO is a signaling molecule which has many physiological and pathological functions, e.g., it enhances the immunosuppressive activities of Tregs [165]. Cals-Grierson and Ormerod [166] reviewed the important role of the UVR-induced generation of NO in inflammation and immunosuppression in the skin. Inflammatory mediators stimulate the expression of IDO1 and enhance the production of KYN and subsequently its metabolites, e.g., 3-hydroxykynurenine (3-HK) and quinolinic acid (QUIN) [167]. QUIN is involved in several pathological states since it has been reported to enhance oxidative stress in the tissues [168]. Furthermore, KYN and KYNA as well as the UVR-generated FICZ stimulate the AhR-mediated transcription “UVR-induced immunosuppression”. Given that the skin is a barrier tissue, the expression of AhR is enriched in the skin, both in keratinocytes and immune cells [133, 169]. AhR has a crucial role in the generation and maintenance of an immunosuppressive state in inflamed tissues [167], as well as in the UVR-induced immunosuppression [131]. AhR factor also exerts both beneficial and harmful effects in the skin in a ligand and tissue-dependent manner [170]. Moreover, the responses induced by AhR factor are dependent on whether the activation is acute or chronic in its nature, i.e., acute responses seem to be protective and adaptive, whereas in chronic inflammatory conditions, AhR signaling promotes premature aging and skin cancers [133]. Currently, the molecular mechanisms driving the photoaging process need to be clarified although it seems that the prolonged presence of an immunosuppressive state has a crucial role in the pathogenesis.

### Is UVR-induced photoaging a proper model of physiological aging?

There are a number of theories on the cause of the chronological aging process and many models of accelerated aging process have been established although it is still far from resolved after several decades of research examining molecular mechanisms. Interestingly, the UVR-induced photoaging in the skin reveals many similar pathological processes to those that are also evident in normal aging. For instance, an accelerated photoaging is associated with DNA damage and telomere shortening in the skin [48, 49, 171]. Both DNA damage and telomere shortening are well-known theories which have been proposed to underpin the aging process across species [172, 173]. Oxidative stress induced by free radicals with aging, a phenomenon also present in UVR-induced photoaging, is one of the oldest aging theories [174]. The UVR-induced alterations in ECM proteins seem to support the garbage accumulation theory of aging [175]. Moreover, there are studies indicating that the exposure of rapamycin, an inhibitor of mammalian target of rapamycin (mTOR), is able to inhibit cellular senescence and even to extend the lifespan of mice [176, 177]. Interestingly, Chung et al. [178] reported that topical treatment with rapamycin reduced the markers of skin senescence and aging in patients exhibiting the clinical signs of photoaging. Accordingly, Qin et al. [179] demonstrated that rapamycin treatment could protect skin fibroblasts from UVB-induced cellular senescence and prevent the appearance of some markers of photoaging. In addition, Xiao et al. [180] reported that metformin, a promising anti-aging drug candidate, inhibited the expression and secretion of inflammatory cytokines from UVB-treated human keratinocytes and protected keratinocytes from apoptotic cell death. They also revealed that the topical administration of metformin in mice was able to inhibit the skin damage evoked by exposure to UVB. Metformin, a potent stimulator of AMP-activated protein kinase (AMPK) signaling, is a well-known inhibitor of several hallmarks of aging [181]. It seems that UVR induces many age-related alterations which can be attenuated with the same anti-aging drugs which are known to be effective against chronological aging.

For over three decades, immunosuppression has been a characteristic hallmark of UVR exposures in the skin. Contact hypersensitivity and the delayed-type of hypersensitivity are simple in vivo assays of cell-mediated immune responses to UVR treatments. Early experiments demonstrated that the UVB-induced immune deficiency was attributable to the presence of suppressive immune cells [182]. Tregs are the major immunosuppressive cells induced by UVR although MDSCs, DCregs, and probably other members of immunosuppressive network are involved in the generation of the immunodeficient state in the skin. Interestingly, the UVR-exposed skin is also able to trigger systemic immunodeficiency “UVR-induced immunosuppression”. A systemic immunodeficiency also occurs in the normal aging process, called immunosenescence [183]. Interestingly, there is convincing evidence that the age-related increase in immunosuppressive activity, attributed to augmented levels of Tregs, MDSCs, and DCregs, is associated with the generation of immunosenescence state with aging [124]. There is substantial evidence that inflammatory states have a major role in the activation of immunosuppressive cells, both in the chronological aging and photoaging. The accumulation of senescent cells with pro-inflammatory properties seems to be a driving force for the generation and maintenance of a chronic inflammatory state which consequently stimulates the counteracting immunosuppressive response. Senescent cells accumulate into both the UVR-exposed skin and chronologically aged tissues [23, 59]. Consequently, the elevated numbers of senescent cells, both immune and non-immune cells, and immunosuppressive cells disturb the homeostasis of aged and photoaged tissues. It seems that cellular senescence and the inflammation-induced immunosuppression stimulate degenerative processes which are far a less similar in both normal aging and photoaging. However, the molecular insults inducing cellular senescence and inflammation are most probably different in the chronological aging and the UVR-induced photoaging.

## Conclusion

UVR exposure is a well-known treatment to induce a local immunosuppression in the skin. Moreover, it is not only a local immune suppressive state, but also the UVR treatment of the skin can induce a systemic immune deficiency in the body. Subsequent investigations have demonstrated that the damages induced by UVR in the skin elicit cellular senescence and inflammation which consequently evoke both local and systemic immunosuppression. In addition to Tregs, it is known that several other immunosuppressive cells also become activated, especially MDSCs and DCregs, probably through the co-activating mechanisms. These observations clearly indicate that the pathological photoaging state in the skin is driven by cellular senescence and chronic inflammation. Interestingly, the chronological aging of tissues also involves cellular senescence and chronic low-grade inflammation. Recent studies have also revealed that the normal aging process also increases the immunosuppressive activity in the immune tissues, the circulation, and even in the peripheral tissues, like the skin [12, 22]. It seems that a persistent inflammatory state, induced by UVR or some other insults, stimulates immunosuppression and promotes premature aging. For instance, there are observations indicating that the chronic inflammation associated with tumors increases immunosuppression and promotes premature local and systemic aging in cancer survivors [184, 185]. Moreover, patients suffering from infection from the human immunodeficiency virus (HIV) display increased immunosuppression and experience a premature onset of age-related morbidities [186]. Several other chronic inflammatory diseases, such as chronic kidney disease (CKD) and chronic obstructive pulmonary disease (COPD), reveal immunosuppression and a premature aging process [187, 188]. On the other hand, the aging process is a major risk factor for cancers and chronic inflammatory states, e.g., infections [189, 190]. Interestingly, UVR treatment and photoaging expose the skin not only to carcinogenesis [73, 105] but they also affect many systemic processes, e.g., they reduce vaccination efficiency [191] and attenuate autoimmunity diseases [113, 192]. It seems that accelerated photoaging and chronological normal aging display similarities driven by the remodeling of the immune system although most probably different molecular mechanisms trigger cellular senescence and chronic inflammation.
